# The tumor suppressor TERE1 (UBIAD1) prenyltransferase regulates the elevated cholesterol phenotype in castration resistant prostate cancer by controlling a program of ligand dependent SXR target genes

**DOI:** 10.18632/oncotarget.1103

**Published:** 2013-07-07

**Authors:** William J. Fredericks, Jorge Sepulveda, Priti Lal, John E. Tomaszewski, Ming-Fong Lin, Terry McGarvey, Frank J Rauscher, S. Bruce Malkowicz

**Affiliations:** ^1^ Department of Surgery, Division of Urology, University of Pennsylvania and Veterans Affairs Medical Center Philadelphia, Philadelphia, PA; ^2^ Department of Pathology and Cell Biology, Columbia University College of Physicians and Surgeons, New York, NY; ^3^ Department of Pathology and Laboratory Medicine, University of Pennsylvania Medical Center, Philadelphia, PA; ^4^ Department of Pathology and Anatomical Sciences, State University New York, Buffalo, NY; ^5^ Department of Biochemistry and Molecular Biology, University of Nebraska Medical Center, Omaha, NE; ^6^ Department of Anatomy, Kirksville Osteopathic Medical School, Kirksville, MO; ^7^ The Wistar Institute, Philadelphia, PA

**Keywords:** TERE1, UBIAD1, prostate cancer, castrate resistant prostate cancer, CRPC, LnCaP, cholesterol, SXR, androgen catabolism, Vitamin K-2

## Abstract

Castrate-Resistant Prostate Cancer (CRPC) is characterized by persistent androgen receptor-driven tumor growth in the apparent absence of systemic androgens. Current evidence suggests that CRPC cells can produce their own androgens from endogenous sterol precursors that act in an intracrine manner to stimulate tumor growth. The mechanisms by which CRPC cells become steroidogenic during tumor progression are not well defined. Herein we describe a novel link between the elevated cholesterol phenotype of CRPC and the TERE1 tumor suppressor protein, a prenyltransferase that synthesizes vitamin K-2, which is a potent endogenous ligand for the SXR nuclear hormone receptor. We show that 50% of primary and metastatic prostate cancer specimens exhibit a loss of TERE1 expression and we establish a correlation between TERE1 expression and cholesterol in the LnCaP-C81 steroidogenic cell model of the CRPC. LnCaP-C81 cells also lack TERE1 protein, and show elevated cholesterol synthetic rates, higher steady state levels of cholesterol, and increased expression of enzymes in the *de novo* cholesterol biosynthetic pathways than the non-steroidogenic prostate cancer cells. C81 cells also show decreased expression of the SXR nuclear hormone receptor and a panel of directly regulated SXR target genes that govern cholesterol efflux and steroid catabolism. Thus, a combination of increased synthesis, along with decreased efflux and catabolism likely underlies the CRPC phenotype: SXR might coordinately regulate this phenotype. Moreover, TERE1 controls synthesis of vitamin K-2, which is a potent endogenous ligand for SXR activation, strongly suggesting a link between TERE1 levels, K-2 synthesis and SXR target gene regulation. We demonstrate that following ectopic TERE1 expression or induction of endogenous TERE1, the elevated cholesterol levels in C81 cells are reduced. Moreover, reconstitution of TERE1 expression in C81 cells reactivates SXR and switches on a suite of SXR target genes that coordinately promote both cholesterol efflux and androgen catabolism. Thus, loss of TERE1 during tumor progression reduces K-2 levels resulting in reduced transcription of SXR target genes. We propose that TERE1 controls the CPRC phenotype by regulating the endogenous levels of Vitamin K-2 and hence the transcriptional control of a suite of steroidogenic genes via the SXR receptor. These data implicate the TERE1 protein as a previously unrecognized link affecting cholesterol and androgen accumulation that could govern acquisition of the CRPC phenotype.

## INTRODUCTION

**Figure 1:**
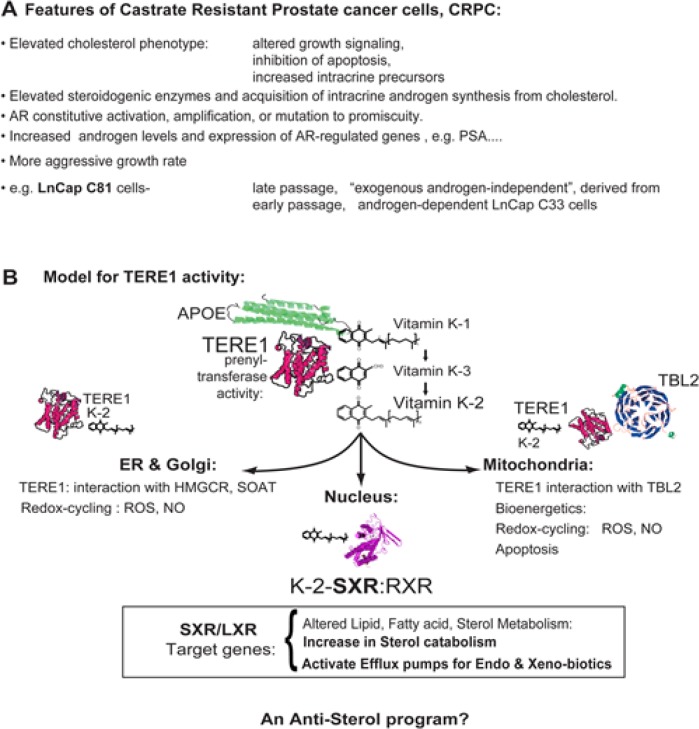
**A.** Features of advanced Castrate Resistant Prostate Cancer cells. The elevated cholesterol phenotype of advanced prostate cancer affects growth signaling, apoptosis and steroidogenesis. The LnCaP cell line C81 was derived from the C33 clone by extended serial passage and has acquired an altered program of gene expression that permits androgen synthesis. We propose this includes a loss of TERE1 expression. This manuscript describes the role of TERE1 and its novel link to *SXR*-mediated mechanisms of regulating sterol accumulation in cells. B. TERE1 at the nexus of cholesterol synthesis, storage, and efflux: Overview of vitamin K-2 effects on cellular metabolism. APOE is a carrier of vitamin K-1, cholesterol, and triglycerides that interacts with TERE1 and is involved in K-1 delivery as well as lipid recycling and efflux. TERE1 converts K-1 to K-2 at multiple locations: golgi, ER, and mitochondria. In ER and golgi TERE1 may interact with *HMGCR* and *SOAT1* thus affect cholesterol synthesis and storage. Based on redox-cyling the K-2 and K-3 quinones may create reactive oxygen species, ROS, and nitric oxide, NO. In mitochondria K-2 plays a role in apoptosis, electron transport and may play a role in mitochondrial bioenergetics in anaerobic environments. TERE1 synthesis of vitamin K-2 creates a potent endogenous activator of the *SXR* nuclear receptor, which traverses to the nucleus with RXR and is a master regulator of endobiotic lipid and fatty acid homeostasis, Phase I and II enzymes and transporters involved in drug metabolism/clearance, and efflux of cholesterol and steroids. In this regard, TERE1 elicits an anti-sterol program that may reverse the elevated cholesterol phenotype of CRPC.

Prostate cancer remains the most common male malignancy and the second most common form of male cancer death [1-3]. The common characteristic of the disease through its clinical progression is androgen receptor activation, which is initially accomplished by systemic androgens. Abrogation of this pathway is a common clinical treatment, which becomes ineffective as tumors overcome castration induced levels of systemic androgens by autocrine stimulation of self produced androgens or androgen receptor activation through mutations (receptor promiscuity to ligands or constitutive activation) [4]. Although the activation of androgen bio-synthetic pathway enzymes in tumor cells has been demonstrated recently [5-9], the metabolism of baseline hormone precursors such as cholesterol are less well understood throughout the progression of prostate cancer. Fig. 1A lists some features of Castrate Resistant Prostate cancers [4, 10, 11]. Both primary tumors and cell line models are characterized by aggressive growth, and elevated levels of intracellular cholesterol [12] which can cause altered growth signaling [13, 14], inhibition of apoptosis [15-19], and provide intracrine precursors for steroidogenesis, resulting in activation of androgen receptor driven target genes associated with proliferation [5-9, 20].

Cellular cholesterol levels are normally highly regulated via a complex interplay between several processes: transport (influx and efflux), de novo synthesis, trafficking, storage, recycling and catabolism to bile acids and steroid hormones [21, 22]. Generally the SREBP transcriptional regulator proteins activate genes for cholesterol synthesis and influx and the LXR and SXR nuclear receptors activate cholesterol efflux; however, both also regulate different aspects of fatty acid metabolism [23]. LXR targets can also be cross-regulated by SXR, the steroid and xenobiotic receptor, or activated by oxysterols derived from the cholesterol pathway or by fatty acids [23-25]. LXR/SXR pathways activate the apo-protein carriers such as APOAI, APOE, and the transporters such as the ATP binding cassette proteins ABC-A1, -G1, -G4, -G5, -G8, and SRBI, through which efflux proceeds to mature HDL [26, 27]. The multiple ways these networks may be dysregulated in the context of tumor cell metabolic reprogramming during progression is not clearly defined.

A reasonable assumption is that during progression either gain or loss of function in oncogenes, or tumor suppressor genes contributes to the elevated cholesterol and steroidogenic phenotype of CRPC [28]. A new candidate for this type of regulation is the *TERE1* gene (aka *UBIAD1*) at chromosome 1p36, first discovered in this laboratory as absent or diminished in a large proportion of prostate, bladder and renal tumors [29-33]. An overview of TERE1 protein function is shown in Fig. 1B. The 338 amino acid TERE1 protein contains ten α-helical trans-membrane domains, a CRAC motif (cholesterol recognition amino acid concensus), and several solution exposed loops that constitute binding interfaces for interacting proteins [32-34]. Protein interaction studies identified APOE, TBL2, HMGCR, and SOAT1 as TERE1-interacting proteins, strongly suggesting a role in cholesterol homeostasis [32-34]. TERE1 sub-cellular distribution encompasses a range of cholesterol regulatory sites including endoplasmic reticulum, golgi, and mitochondria. Reconstitution of TERE1 expression in bladder, renal, and prostate tumor cell that have silenced the endogenous gene results in strong inhibition of tumor cell growth. Remarkably, germline mutations in TERE1 cause a rare disease of elevated corneal cholesterol and lipid deposition called Schnyder's Corneal Dystrophy, SCD [31, 35], and these mutations alter binding to APOE, HMGR, and TBL2 [32-34]. However, the mechanisms by which TERE1 modulates cholesterol homeostasis via its direct protein interaction with mediators of synthesis (HMGR), storage (SOAT1), and efflux (APOE) are not yet well defined and undoubtedly complex.

The recent identification of TERE1 as the prenyltransferase required for vitamin K-2 biosynthesis provides a basis for understanding its multi-factorial influence on the cell, including effects on steroidiogenesis. TERE1-mediated synthesis of vitamin K-2 clearly affects lipid metabolism, redox balance and mitochondrial function [36]. Numerous studies describe tumor cell growth inhibition based on the redox-cycling and alkylating properties of vitamins K-2 and K-3, suggesting a basis for TERE1 tumor suppressor activity [37-39]. Another dimension of TERE1 activity relates to its mitochondrial localized activity: its Drosophila homolog, *heix*, was found to enhance mitochondrial electron transport and ATP production [40], a finding consistent with the bioenergetics of K-2 in anaerobic bacteria and mitochondria [41, 42]. We recently reported immuno-electron microscopic localization of TERE1 in mitochondria along with TBL2, and the effects on mitochondrial trans-membrane potential, and the generation of ROS/RNS [33]. TERE1 can also the expression of nuclear genes: its product, vitamin K-2 is a potent ligand for activating the SXR nuclear hormone receptor and hence SXR target genes [37, 38], many of which are well known to encode enzymes in the *de novo* cholesterol biosynthetic pathway. We thus investigated TERE1 function as a modulator of the elevated cholesterol phenotype of CRPC [25, 36, 43-46] by focusing on the ability of the TERE1 product, K-2 to activate SXR target genes which regulate sterol accumulation [47]. Our findings point to a key role for TERE1 in modulating cholesterol and steroid accumulation in prostate tumors as a means of regulating growth and progression of this neoplasm.

## RESULTS

### TERE1 expression in metastatic prostate cancer

**Figure 2:**
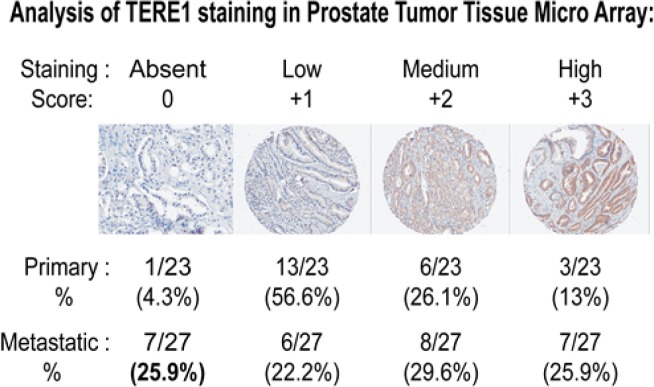
Reduced TERE1 staining in prostate carcinoma tissue micro-array The representative labeling index groups (Absent, Low, Medium, and High) based on the intensity of staining x percentage of tumor cells staining with anti-TERE1 antibody (affinity purified chicken polyclonal against amino acids 229-242). TERE1 protein is reduced (absent or low) in ~ half of primary and metastatic human prostate carcinoma specimens. TERE1 expression was absent in 25% of metastatic lesions compared to 4% of primary specimens suggesting loss of TERE1 expression may be a marker for advanced disease

To determine the frequency of TERE1 alteration in human prostate cancers we conducted an immuno-histochemical analysis using a custom human prostate tumor microarray (TMA) to examine TERE1 expression in primary carcinoma compared to metastatic specimens. The results of 23 primary and 27 metastatic cancer specimens using a well defined chicken anti-TERE1 (229-242) antibody [33] are summarized in Fig. 2. Overall TERE1 staining was heterogeneous. The most obvious change was observed as a loss of staining in the metastatic group. TERE1 staining was absent in a quarter of the metastatic specimens (~26%), in contrast to only a minority of cases (4.3%) of primary specimens. For both primary and metastatic, over 50% of specimens showed complete absence or low levels of staining; hence, a reduced TERE1 expression may represent a significant phenotype in prostate cancer.

### Endogenous TERE1 expression and cholesterol in C81 cells

**Figure 3:**
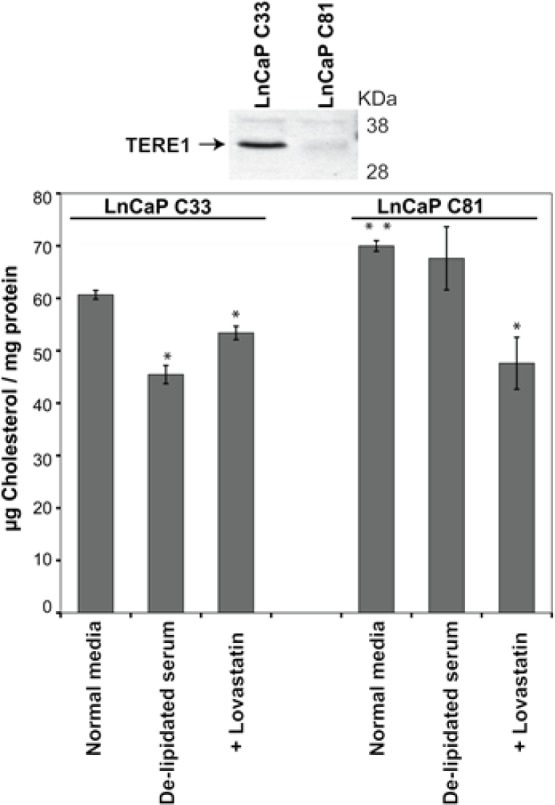
Endogenous TERE1 expression is reduced and cholesterol is elevated in the LnCaP C81 cell model of CRPC The LnCaP prostate cancer cell line C81 serves as a model for the Castrate Resistant Prostate Cancer phenotype based on its steroidogenic potential. C81 was derived from the parental C33 clone which is not steroidogenic. Immunoblot analysis with goat anti-TERE1 antibody showed that C81 expresses a reduced level of TERE1 and has a 17% elevated cholesterol level. In lipid free growth media, C33 cholesterol levels are significantly reduced but C81 level remains unchanged, suggesting C33 is more dependent on transport and C81 more dependent on synthesis and/or retention. The cholesterol level of C81 was reduced by lovastatin (10μM) to a greater extent than C33 suggesting that C81 cells have a greater potential for cholesterol synthesis.

The LnCaP prostate cancer cell subline C81 was derived from its parental cell line, C33 and is a widely accepted model for the CRPC phenotype based on its ability to become steroidogenic when grown in hormone free conditions (5% charcoal stripped serum) [7]. We compared the endogenous TERE1 levels in the C33 and C81 LnCaP clones and found C81 to express a significantly reduced level of TERE1, Fig. 3. Total cholesterol levels of cell lysates were measured using the well-established Amplex Red fluorometric assay relative to a dilution series of cholesterol standards using equal amounts of protein [48]. When we measured the total cholesterol levels in C81 cell lysates we found they were elevated by 17% compared to C33 from cells grown in under full serum conditions (5% FBS). However, when cells were grown in lipoprotein-depleted FBS (which limits import of serum derived cholesterol), the C33 cells showed a significant reduction (25%) in cholesterol, suggesting a greater reliance on uptake rather than synthesis. In contrast, the cholesterol level in the C81 cells was only slightly reduced by growth in lipoprotein-depleted FBS, suggesting they may have an elevated rate of cholesterol synthesis. When the cells were cultured in the presence of lovastatin (10μM) to inhibit endogenous cholesterol synthesis, the cholesterol level of the C81 clone, was reduced (by 35%) to a greater extent than C33 (~10% reduction), suggesting that C81 cells have a greater potential for cholesterol synthesis. Overall this suggests a correlation between TERE1 levels and cholesterol in the C33 and C81 cells, which is consistent with their reported steroidogenic potential [7].

### Elevated cholesterol synthesis

**Figure 4:**
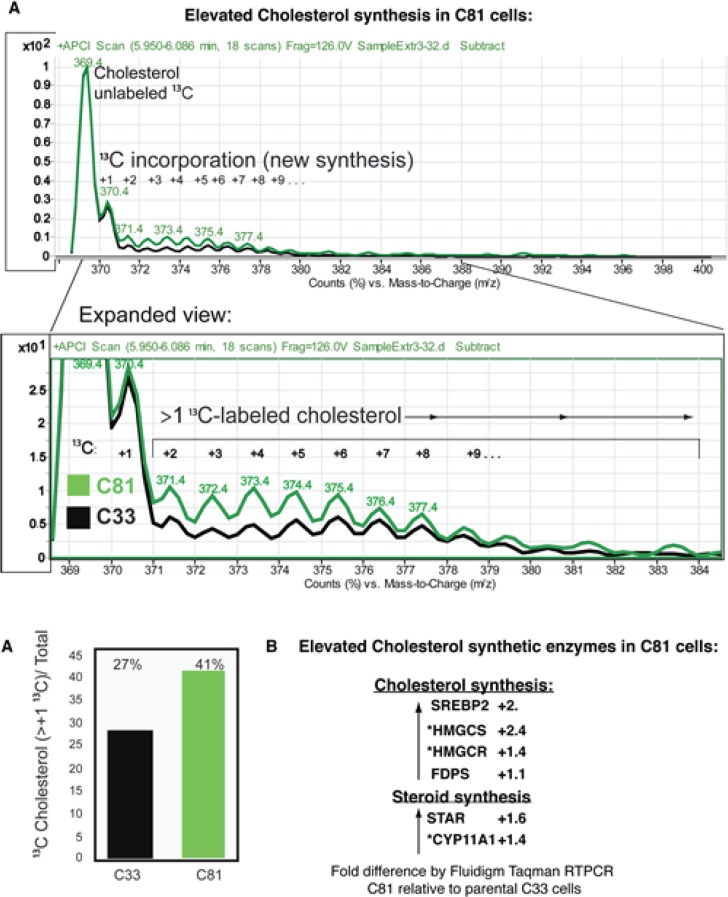
LnCaP C81 cells have elevated cholesterol synthesis 4A. We examined cholesterol synthesis in C33 and C81 cell cultures via LC/MS isotopomer analysis after incorporation of ^13^C-acetate. The sum of the areas of ^13^C-labeled-isotopomers with m/z of 371.4-399.4 co-eluting with the cholesterol peak, divided by the total amount of cholesterol isotopomers, was used as a measure of cholesterol synthesis. C81 cell lysates showed a 52% increase in newly synthesized cholesterol compared to C33 cells. 4B. Fluidigm RT-PCR analysis of C81 transcripts relative to C33 revealed elevated expression of several enzymes involved in cholesterol synthesis and mobilization of cholesterol to mitochondria. Those genes with an asterix* are established *SXR* target genes.

The elevated level of total cholesterol in C81 cells led us to examine cholesterol synthesis via isotopotomer analysis using incorporation of ^13^C-acetate and LC/MS analysis, Fig. 4A. The chromatographic peak for cholesterol was identified using a deuterated-cholesterol (D6-cholesterol) as an internal standard. The sum of the areas of ^13^C labeled-isotopomers with m/z of 371.4-399.4 co-eluting with the cholesterol peak, divided by the total amount of cholesterol isotopomers, was used as a measure of cholesterol synthesis. The lysates from C81 cells showed a 52% increase in the amount of newly synthesized cholesterol compared to C33 cells. To determine the underlying causes of increased cholesterol synthesis we conducted an RTPCR analysis of expression of several enzymes involved in cholesterol synthesis and mobilization to mitochondria as a first step in steroid synthesis (Fig 4B). We found that the expression of *SREBP2, HMGCS, FDPS, HMGCR, STAR*, and *CYP11A1* were all elevated in C81 relative to C33 cells. This is consistent with the elevated cholesterol synthesis that we observe.

### Increased expression of TERE1 protein reduces cellular cholesterol levels

**Figure 5:**
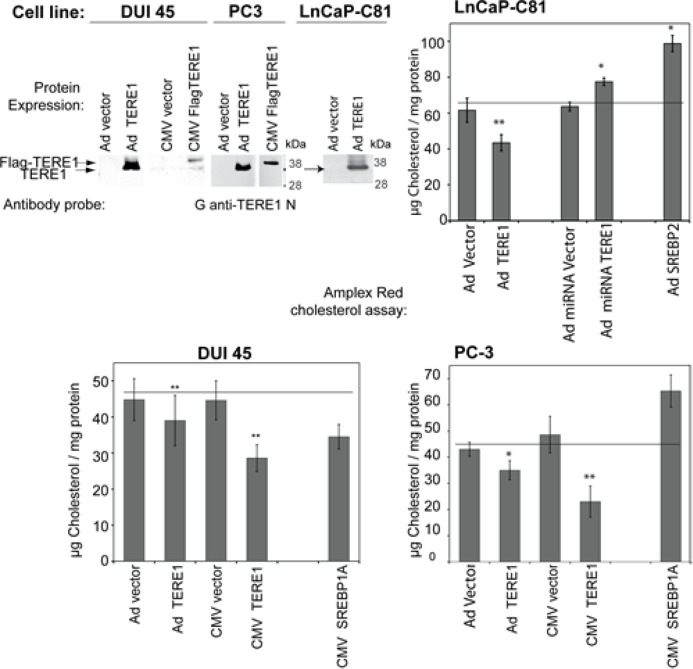
TERE1 protein expression decreases the cellular cholesterol levels in PC-3, DUI45 and LnCaP-C81 prostate cancer cell lines We expressed TERE1 proteins in PC-3, DUI45 and LnCaP-C81 cells via adenovirus transduction or transfection and confirmed expression of the ~37 kDa TERE1 proteins via immunoblots. Cholesterol was measured using the Amplex Red assay. Ectopic expression of TERE1 protein for 72 hours results in a reduced level of cellular cholesterol compared to untreated or Ad-*LACZ* Vector controls. MiRNA-mediated reduction of TERE1 results in an elevation of cellular cholesterol in LnCaP C81 cells.

TERE1 protein levels were assessed in two additional well-studied prostate cancer cell lines originally derived from metastases, DUI45 and PC3, to see if reduced levels of TERE1 in cell lines might be a feature in common with some metastatic prostate cancer specimens. Endogenous TERE1 at ~37 kDa was barely detectable in either of the cells (Fig. 5). After transfection of TERE1 mammalian expression plasmids or adenovirus infection we easily confirmed ectopic expression of TERE1 in LnCap C81, PC-3, and DUI45 cell lines and then measured total cell-derived cholesterol. As a positive control for genes that influence cholesterol levels we transfected an expression plasmid for constitutively active SREBP1a or SREBP2. In these cells we observed an increased cholesterol level with PC-3, and C81, respectively, as would be expected. Elevated expression of TERE1 protein in DUI45, PC-3, or LnCaP C81 cells caused a significantly reduced intracellular cholesterol levels compared to control vector. Knockdown technology was used to reduce the endogenous cellular level of the TERE1 in C81 cells. Significantly, the miRNA specific for TERE1 caused a 15% increase in cholesterol level of LnCaP-C81 cell lysates compared to those transduced with a reference scrambled miRNA control. Thus under these conditions, altered expression of TERE1 can modulate cellular cholesterol levels [32, 33, 49]. As a precursor to steroid synthesis, cholesterol has important implications for prostate cancer and the loss of TERE1 expression may be one of the changes acquired during progression to the castrate resistant phenotype of advanced prostate cancer.

### TERE1 expression is inducible in LnCaP cell clones

**Figure 6:**
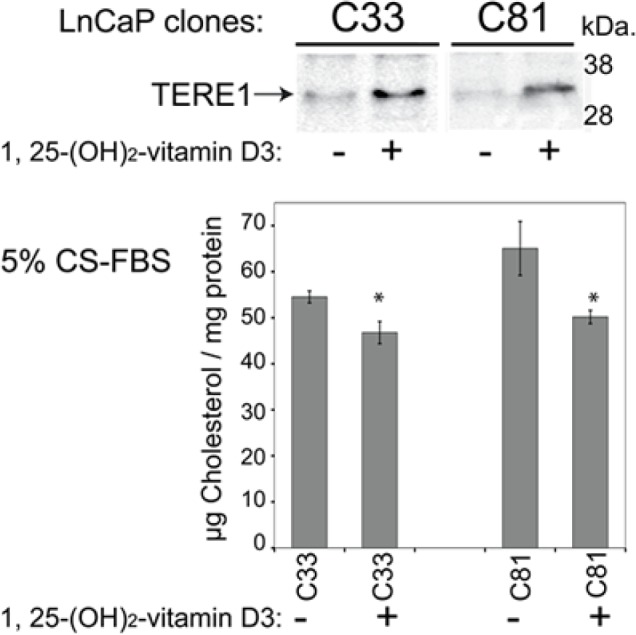
Endogenous TERE1 expression is inducible in LnCaP cell clones Treatment of both C33 and C81 LnCaP cell clones with 1,25-dihydroxy vitamin D3 (100 nM, for a period of 6 days) increased TERE1 protein levels and decreased cell cholesterol.

The presence of vitamin D response elements (VDREs) within the TERE1 promoter [50], and the previously established inhibitory effects of 1,25-(dihydroxy)-vitamin D3 on LnCaP cell growth [51, 52] led us to examine the inducibility of endogenous TERE1 expression and the resultant effects on cholesterol in LnCaP prostate cancer cell clones (Fig. 6). Treatment of cells with 1,25-(dihydroxy)-vitamin D3 (100 nM, for a period of 6 days) increased TERE1 protein levels and decreased cell cholesterol in both LnCaP cell clones, although C81 was more sensitive to the cholesterol reduction than C33. This further demonstrates that TERE1 expression levels influence cholesterol levels. Moreover, these data suggest the possibility that Vitamin D status in humans may affect TERE1 levels and suggest a possible therapeutic use of vitamins D and K2 in androgen-independent prostate cancer.

Overall our studies have found that either exogenous or endogenously induced TERE1 expression can lead to changes in cellular cholesterol in prostate cancer cell lines. Given the established mechanistic connection between vitamin K-2, the product of TERE1 prenyltransferase activity, and K-2's role as a ligand for the *SXR* nuclear receptor, a master regulator of sterol metabolism and homeostasis, we next investigated *SXR* target gene expression in the C33 and C81 cell lines.

### SXR target gene repression in C81 cells

**Figure 7:**
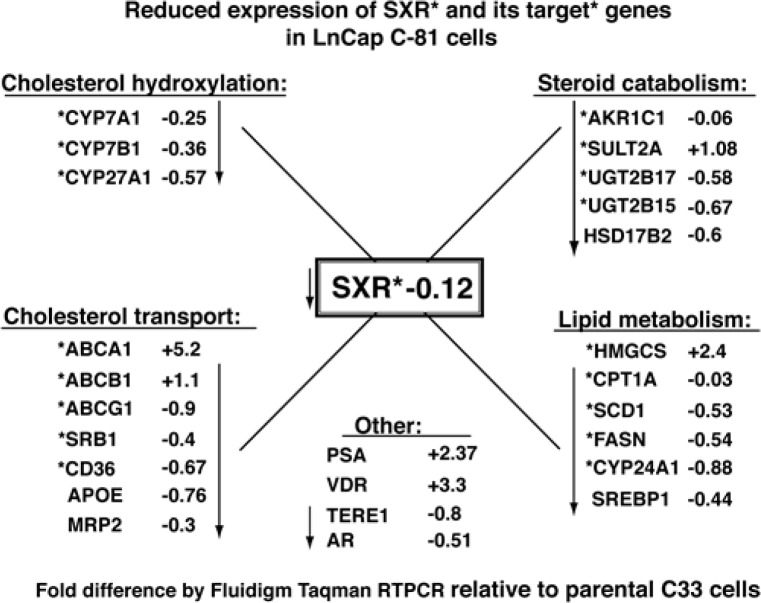
LnCaP C81 cells exhibit reduced expression of *SXR* and *SXR* target genes involved in cholesterol and steroid catabolism Fluidigm RT-PCR Taqman assays were conducted to compare expression of *SXR* target genes in C33 and C81 cells. The genes are sorted into functional categories and established *SXR* target genes are indicated with an asterix *. The fold-differences in gene expression of C81 cells are depicted as a ratio relative to C33 with a “+” for fold-activation and with a “-” for fold-repression. *SXR* and 14 of 18 *SXR* target* genes are repressed in C81 cells. Repression of this suite of *SXR* target genes represents a significant component of the CRPC elevated cholesterol phenotype of LnCaP C81 cells by virtue of the combined potential to preserve sterols from efflux and catabolism.

The steroid and xenobiotic nuclear receptor, *SXR*, has roles in the regulation of endobiotic homeostasis (sterols, lipids) and regulation of transporters involved in xenobiotic clearance [25, 43]. We assembled a comprehensive array of established target genes of *SXR*, of *LXR* (which can be cross-regulated) and several genes involved in the synthesis and catabolism of cholesterol and androgens and tested their levels in the cell lines described above [23, 25, 53-58]. The diagram in Fig. 7 summarizes our findings that depict the fold-difference in gene expression of C81 cells as a ratio relative to C33 cells. The differences are depicted with a “+” for fold-activation and with a “-” for fold-repression. Values were normalized as described in methods. Expression of genes that are established *SXR* target genes are indicated with an asterix*. The most immediate observation is that in C81 cells, the *SXR* gene itself is expressed at 12% of the levels seen in C33 cells and that 14 of 18 *SXR* target genes also show lowered levels. Looking more deeply at the functions of these genes the first category of *SXR* targets are all involved in cholesterol hydroxylation that form the oxysterol efflux forms of cholesterol (CYP7A1*, CYP7B1*, and CYP27A1*). The next set includes cholesterol efflux and transport proteins (ABCB1*, ABCG1*, SRB1*, CD36*, *APOE*, and MRP2) all of which are at lower levels in C81 cells. TERE1-mediated reductions in expression of these two sets of genes could contribute to the elevated cholesterol observed in C81 cells via the sum of multiple mechanisms of accumulation. (In contrast to repression of most efflux mechanisms, we do observe that the ABCA1* cholesterol transporter is activated in C81, underscoring the cell's necessity to maintain an efflux mechanism to keep cholesterol levels below a toxicity threshold.) Another set of repressed genes play a role in steroid catabolism (AKR1C1*, UGT2B17*, UGT2B15*, and *HSD17B2*) and includes several *SXR* targets. The expected effect would be to stabilize androgen production and is consistent with the steroidogenic potential of C81. We also note that the AR target gene PSA is also elevated by more than two-fold. (Under full serum growth conditions steroidogenesis would not be expected to take place, hence, we also observed repression of *SRD5A1*, AKR1C3*, and CYP17A1*, data not shown). Finally, we see a subset of classic *SXR* target genes involved in lipid and fatty acid metabolism is also repressed (HMGCS*, CPT1A*, SCD1*, FASN*, CYP24A1*, and *SREBP1*). Overall, in addition to induction of cholesterol synthesis genes, it appears that repression of *SXR* and its target genes is a significant factor in the C81 CRPC phenotype by virtue of its potential to preserve sterols from efflux and catabolism.

### TERE1 or exogenous vitamin K-2 switches on an anti-sterol suite of SXR target genes

**Figure 8:**
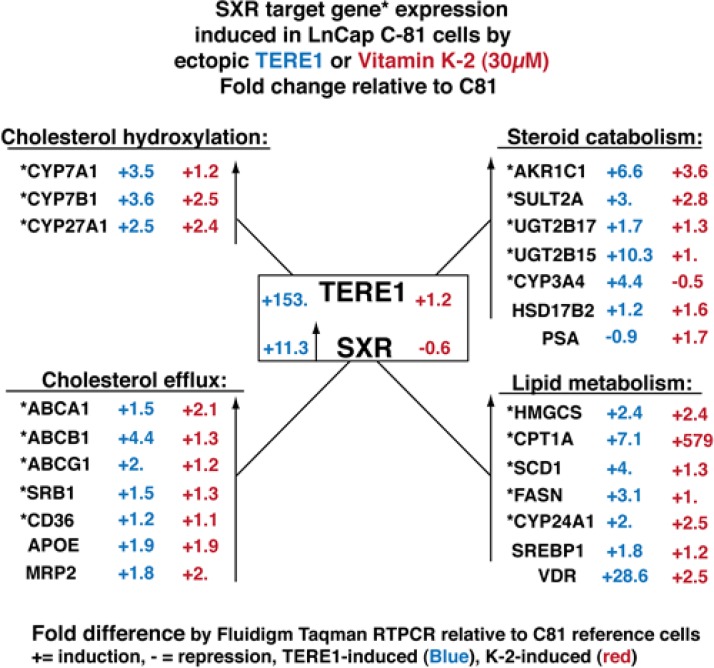
Ectopic TERE1 or Vitamin K-2 reactivate an anti-sterol suite of *SXR* target genes in C81 LnCaP cells involved in cholesterol efflux and steroid catabolism Fluidigm RT-PCR Taqman assays were conducted to evaluate expression changes in C81 cells after ectopic Ad-TERE1 expression or vitamin K-2 (30μM) treatment. The fold-differences in gene expression of the LnCaP cells: C81-TERE1 (in blue), or C81-K-2 (in red), is depicted as a ratio relative to C81 cells with a “+” for fold-activation and with a “-” for fold-repression. TERE1 /K-2 activated *SXR* in C81 cells and each of the *SXR* target* genes tested in this study. Activation of this suite of *SXR* target genes represents a significant anti-sterol gene expression program with potential to oppose the CRPC elevated cholesterol phenotype of LnCaP C81 cells by virtue of its combined potential to efflux cholesterol and catabolize androgens.

**Figure 9:**
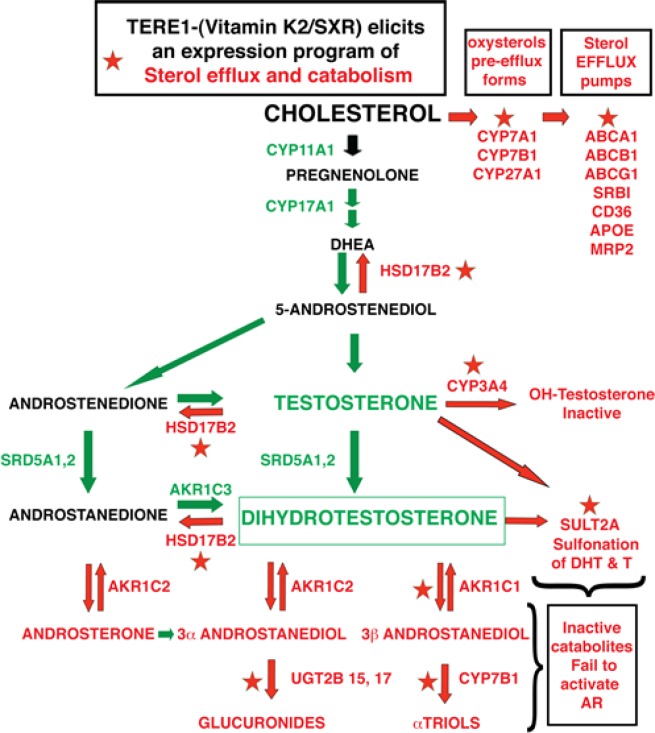
Overview of the Anti-Sterol targets activated by TERE1/vitamin K-2 in C81 LnCaP cells TERE1 activates *SXR* target genes (indicated by stars) that hydroxylate cholesterol (*CYP7A1, CYP7B1, CYP27A1*) to form oxysterol efflux forms of cholesterol, and activates a panel of transporters (*ABCA1, ABCB1, ABCG1, SRBI, CD36, APOE, MRP2*) that promote cholesterol efflux from the cell. Together these would serve to diminish cholesterol, the major precursor for androgen synthesis. TERE1 also turns on a program of androgen catabolic enzymes with the following activities: *CYP3A4* hydroxylates testosterone thus facilitating efflux, *SULT2A* sulfonates DHT to a form that does not activate the Androgen receptor, *HSD17B2* converts Testosterone to estrogen, *AKR1C1* catabolizes DHT by reduction to the inactive 3b andostanediol, and *UGT2B15* and *UGT2B17* glucuronidate the 3a andostanediol catabolite of DHT thus preventing its back conversion to DHT. Overall this suite of TERE1 /K-2/*SXR*-driven activities executes an anti-sterol program that opposes the CRPC phenotype.

Next we extended our RT-PCR analysis to investigate the potential for TERE1 or vitamin K-2 to elicit a change in *SXR* target gene expression. We analyzed expression from parallel sets of C81 cells that had received ectopic Ad-TERE1 expression virus for 72 hours (C81-TERE1) or dosing with vitamin K-2 (30μM) overnight (C81-K-2). Interrogating the custom array of genes by RTPCR revealed a dramatic reversal in expression of *SXR* and its target genes. In Fig. 8 the fold expression change of the C81 cells transduced with TERE1, or treated with K-2 is depicted relative to the C81 vector control. First, *SXR* expression is increased 11.3-fold by TERE1 expression. Consistently, we see that expression of *SXR* target* genes (18/18 genes) is increased by TERE1 and many of the same genes are also increased by Vitamin K-2 across each of the 4 groups. TERE1 or K-2 treatments activate the set of classical *SXR* target genes that regulate fatty acid and lipid metabolism (HMGCS*, CPT1A*, SCD1*, FASN*, CYP24A1*, *SREBP1* and *VDR*). Highly relevant to the reversal of the elevated cholesterol phenotype of CRPC, we observed increased expression of cholesterol hydroxylation enzyme and cholesterol efflux genes that would significantly reduce the cholesterol levels in C81 cells and likely account for the TERE1-mediated cholesterol reduction we observe and potentially limit the supply of sterol precursors for androgen synthesis. Also evident is a dramatic increase in expression of androgen catabolic genes (AKR1C1*, SULT2A*, UGT2B17*, UGT2B15*, CYP3A4*, and *HSD17B2*). The predicted net effect of alterations in the abundance/activity of these enzymes would be the efflux, inactivation, or breakdown of DHT and its precursors, which could potentially decrease AR activation driven proliferation (Fig. 9).

Overall, these data demonstrate that TERE1 expression can lead to regulation of *SXR* target genes involved in lipid metabolism and is consistent with the hypothesis that this is due to activation of *SXR* by TERE1-mediated synthesis of K-2 [25, 36, 46].

## DISCUSSION

### >Elevated Tumor cell Cholesterol and Prostate Cancer

Androgen ablation therapy has been limited by the emergence of a “castrate resistant phenotype” in most advanced prostate cancer patients. Recent work demonstrates that androgen dependence may not be exhausted by systemic castration and inhibition of extragonadal sources of androgen. The demonstration of *de novo* synthesis of androgens from cholesterol precursors and from progesterone in LnCaP cell models of castrate resistant prostate cancer, CRPC, underscores the critical role of intracrine mechanisms as a potential driving force in cancer progression in the castrate state [5-9]. The focus on cholesterol as an intracrine steroid precursor of progesterone in mitochondria is further relevant in light of additional studies showing that mitochondrial cholesterol levels are frequently elevated in prostate cancers and can interfere with membrane associated signaling complexes required to trigger early mitochondrial apoptotic events, and to regulate important pathways governing cell proliferation [13, 16, 19, 59-61]. The very signaling pathways now recognized as being cholesterol-dependent: *AR, TGFβ, EGF, MAPK/ERK, AP-1*, and *AKT*, are well characterized as prostate cancer relevant [62, 63]. Thus in addition to its role as a steroid precursor, cholesterol has emerged as a key molecule to prostate cancer progression [2, 64, 65].

### TERE1 expression in prostate cancer specimens and cell lines

Our objectives in this study were to gain further insights into the role of the TERE1 protein in the elevated cholesterol phenotype of advanced Castrate Resistant Prostate Cancer, CRPC. Our initial interest was precipitated by our earlier demonstration that TERE1 message was reduced in ~60% (18 of 30) of human prostate cancer specimens [29, 30]. We have also found similar TERE1 expression reduction in renal clear cell cancers, another example of a tumor with an elevated cholesterol phenotype and in bladder cancer [32, 33, 49]. With this prostate cancer study, our TERE1 immunohistochemical analysis found TERE1 staining was reduced or completely absent in over half of 50 primary and metastatic specimens. TERE1 expression was absent in 25% of metastatic lesions compared to 4% of primary specimens, suggesting further loss of TERE1 during progression. Hence, a reduced expression of the TERE1 protein occurs with sufficient frequency to potentially affect a significant number of individuals.

We also found a reduced TERE1 protein expression level in the LnCaP C81 cell model of advanced CRPC, relative to the parental LnCaP-C33 clone from which it was derived. Low levels were similarly observed in both PC-3 and DUI45 cell lines. Taken together these observations suggest that TERE1 expression may represent a liability to prostate tumor cell metabolism during progression.

### TERE1, Cholesterol and SXR target gene analysis in LnCaP-C81 cells

The relevance of TERE1 loss to the elevated cholesterol phenotype of advanced prostate cancer is further based on previous studies from our laboratory and others that have demonstrated the role of TERE1 as a central modulator of cholesterol homeostasis, at the nexus of cholesterol synthesis, storage and efflux. TERE1 interacts with: *APOE*, a cholesterol, triglyceride, and vitamin K transport protein, *HMGR*, a master cholesterol biosynthetic regulatory enzyme, and *SOAT1*, a regulator of cholesterol storage [32-34, 66]. By virtue of its direct role in vitamin K-2 synthesis, TERE1 activates the *SXR* nuclear receptor, a master regulator of lipid, sterol and xenobiotic homeostasis, and drug metabolic enzymes and transporters [25, 36, 45, 46]. Upon binding to a diverse number of ligands, SXR heterodimerizes with RXR and cross-regulates target genes involved in oxidative, peroxidative, and reductive metabolism of many endogenous and xenobiotic substrates [25, 67]. It exhibits promiscuous binding to DR-3, DR-4, DR-5, ER-6, and ER-8 response elements in target gene promoters, including those with well-established roles in modulation of cellular cholesterol efflux such as LXR [23, 54]. The relevance of TERE1-mediated vitamin K prenyltransferase activity to SXR activation is further substantiated by a recent study showing that vitamin K-2 analogs with increasing number of prenyl groups were more potent ligands for SXR-mediated transcriptional activity [68, 69]. Epidemiological studies have also noted an inverse association between vitamin K-2 intake and the risk of prostate and other cancers [70]. SXR regulation of endogenous sterol metabolites makes this nuclear receptor a high profile target for hormone dependent tumors [47].

Consequently, we have focused our investigation on the correlation of TERE1 with cholesterol levels and SXR target genes using the LnCaP-C81 cell model of the CRPC phenotype [7]. In comparison to the non-steroidogenic C33 clone, C81 cells had increased expression of cholesterol synthetic enzymes (SREBP2, HMGCS, FDPS, and HMGCR), and an elevated cholesterol synthesis measured via ^13^C acetate incorporation into newly synthesiszed cholesterol. Equally significant is the diminished expression of the SXR target gene axis that includes a panel of cholesterol hydroxylation (CYP7A1*, CYP7B1*, and CYP27A1*) and efflux proteins (ABCB1*, ABCG1*, SR-B1*, CD36*, *APOE*, and MRP2) as well as androgen catabolic enzymes (AKR1C1*, UGT2B17*, UGT2B15*, and *HSD17B2*) and several classic *SXR* target genes involved in lipid and fatty acid metabolism (HMGCS*, CPT1A*, SCD1*, FASN*, CYP24A1*, and *SREBP1*) (Figs. 7 and 9). Apparently the C81 CRPC cells use multiple strategies to preserve the sterol precursors and active androgens that their AR-driven proliferation is dependent on. In contrast to our findings in LnCaP-C81 cells, others have reported that ABCA1 is silenced by promoter hypermethylation in the parental LnCaP cells [71]. This may account for its apparent increase in C81 when evaluated relative to parental LnCaP cell. It also makes sense that the sterol efflux pump ABCA1* remains actively expressed as a way to focus regulation of potentially toxic sterol levels to a smaller set of pumps for tighter control. All of these changes would serve to preserve sterols from efflux and catabolism and contribute to the elevated cholesterol phenotype of CRPC.

### Vitamin K2/SXR and Cholesterol and Lipid Metabolism

Our investigation of the role of TERE1 in prostate cancer supports the hypothesis of a vitamin K-2 mechanism for TERE1-mediated cholesterol modulation. Upon restoration of TERE1 levels in C81 cells via ectopic expression (Fig. 8. and 9) we first noticed an increase in SXR expression and although the exact mechanism of SXR induction was not studied here, it is consistent with the known response of the SXR promoter to changes in the metabolic fluxome [72]. In addition, we also see the expected activation of the complete SXR axis, consistent with the K-2 activation mechanism of SXR nuclear signaling and the SXR increase. In most cases there is concordance between TERE1 and K-2 in the correlation with activation of SXR and other targets, although a curious exception is the SXR mRNA itself, which was not increased by K-2. The set of genes that were repressed in the C81 basal state are now all switched on by TERE1: these include the cholesterol hydroxylation (CYP7A1*, CYP7B1*, and CYP27A1*) and efflux proteins (ABCB1*, ABCG1*, SR-B1*, CD36*, *APOE*, and MRP2). This change in gene expression most likely accounts for the TERE1-mediated cholesterol reduction we observe. Also expected is the increased expression of *SXR* target genes that regulate fatty acid and lipid metabolism (HMGCS*, CPT1A*, SCD1*, FASN*, CYP24A1*, *SREBP1*), thus reinforcing that a K-2/SXR mechanism is in effect.

Our findings are consistent with several reports of SXR activating one or another of these targets, but this is the first demonstration of TERE1-mediated endogenous K-2 synthesis switching on a suite of SXR targets in prostate cancer cells. A K-2/SXR mechanism was demonstrated to increase expression of ABCB1 involved in cholesterol efflux in osteoblastic cells [45, 73, 74]. ABCB1 was also induced by the SXR agonist rifampicin in breast cancer cells [75, 76]. Menadione (K-3) has also been shown to activate another cholesterol transporter, ABCG2 in HEK-293 cells. Impaired LXR-mediated regulation of ABCA1 and ABCG1 involved in cholesterol efflux has been proposed as a defect in LnCaP and PC3 prostate cancer cell lines that contributes to cholesterol elevation [77]. The inhibition of LnCaP cell xenografted tumors in mice via LXR agonists has been previously associated with elevation of ABCA1 [78]. We also show that TERE1 protein expression can be induced endogenously via 1,25-(dihydroxy)-vitamin D3 and result in a decrease in cellular cholesterol. This is consistent with the presence of VDREs in the TERE1 promoter, the known expression of the VDR in LnCaP cells [50, 79], and early reports that calcitriol can reduce cholesterol and inhibit HMGCR activity in cultured cells [80]. The well-known correlation between vitamin D deficiency and prostate cancer has evolved over many years of epidemiological, cell and molecular studies [81]. The active vitamin D metabolite, 1,25-(dihydroxy)-vitamin D3, calcitriol, has been demonstrated to slow the progression of prostrate cancer to advanced disease and inhibit growth of many different prostate cancer cell lines. We noted that 1,25-(dihydroxy)-vitamin D3 caused a potent induction (>500-fold, data not shown) of SULT2A which is an important mechanism for androgen inactivation [82]. In addition to cholesterol, studies have shown that elevated ABCB1 also effluxes DHT in LnCaP prostate cancer cells, which would further serve a role in anti-androgen accumulation [83].

### TERE1 /Vitamin K2/SXR and androgen catabolism

The next critical dimension of TERE1-mediated expression changes relevant to the CRPC phenotype concern the set of anti-androgenic targets of *SXR* that have diminished activity in LnCaP C81 cells but are turned on by TERE1 (AKR1C1*, SULT2A*, UGT2B17*, UGT2B15*, CYP3A4*, and *HSD17B2*). Our findings are supported by several reports highlighting the relevance of these targets in androgen catabolism [47]. The AKR1C1 and AKR1C2 enzymes are known to play a role in catabolism of DHT (Fig 9.), and reduce androgen-dependent growth [84]. AKR1C1 converts 5α-dihydroxytestosterone to 3β-androstanediol. AKR1C2 converts 5α-dihydroxytestosterone and androsterone to 3α-androstanediol. Both of these catabolic products are unable to activate the androgen receptor [84, 85]. Impaired catabolism by both AKR1C1 and AKR1C2 was found to be a critical step in androgen signaling [86]. The vitamin K-2/SXR mechanism was proven to activate expression of AKR1C1 and AKR1C2 in osteoblastic cells [73], but TERE1/K-2-mediated activation of SXR has not been previously studied in prostate cancer. SULT2A1, a DHEA sulfotransferase, known to cause sulfonation of androgens and reduce androgenic activity is another target activated by TERE1/SXR. LXR/SXR agonists were previously shown to activate SULT2A in LnCaP cells and inhibit testosterone stimulated proliferation and PSA reporter activity [82]. TERE1 activation of *CYP3A4* is consistent with reports of *SXR*-induced *CYP3A4*, which hydroxylates testosterone and progesterone leading to inactive metabolites and resulted in inhibition of androgen-dependent growth of human LAPC-4 prostate cancer cells [43, 47]. The UDP-glucuronosyl transferase enzymes, UGT 2B15 and 2B17 are expressed in prostate and known to inactivate testosterone, DHT, and 3α androstanediol by glucuronidation [87]. UGT 2B15 and 2B17 are activated by FOXA1 in LnCaP cells and SXR is known to interact with FOX homologs and cross regulate their targets.

The predicted net effect of activity of these TERE1-induced enzymes would be the efflux, inactivation, or breakdown of DHT and precursors, which would decrease AR activation driven proliferation (Fig. 9). Recently, a novel role for SXR as a direct repressor of androgen receptor signaling in DUI45 and LnCaP cell lines was identified. Coimmunoprecipitation and colocalization evidence suggested a model in which SXR agonists and AR antagonists could induce binding of SXR with the AR resulting in SXR-mediated repression of AR targets [88]. This suggests the possibility that TERE1/K-2/SXR may directly inhibit AR signaling in addition to the effects reducing cholesterol and catabolizing AR-activating androgens.

In summary, we have established a TERE1-negative expression phenotype to 50% of human prostate tumor specimens, correlated cholesterol synthesis and accumulation in prostate cancer cell lines with TERE1 exogenous or endogenous expression. Furthermore, we have defined an anti-sterol suite of *SXR* target genes promoting cholesterol efflux and androgen catabolism that are repressed in the LnCaP-C81 cell model of CRPC, but are switched on by TERE1 expression and vitamin K-2. The loss of TERE1 expression may be a defect that tumors use to uncouple vitamin K-2 from nuclear SXR signaling to permit elevation of cholesterol and facilitate androgen driven progression. We believe TERE1 holds promise as a new determinant in cholesterol-mediated progression of prostate cancer and an exploitable modulator of androgen metabolism to oppose CRPC.

## MATERIALS AND METHODS

### Cell lines and antibodies

The LnCap C33 and C81 cell clones were generously provided by and cultured as specified by Dr Ming-Fong Lin (University of Nebraska Medical Center). The PC3 and DUI45 cell lines were obtained from the American Type Culture Collection (Manassas, VA) and grown according to supplier's instructions. Goat anti-TERE1 antibodies were obtained from Santa Cruz. Affinity purified polyclonal antibodies against specific peptide antigens of the human TERE1 (amino acids 229-242) have been previously described [32, 33, 49].

### Immunohistochemistry

Five-micron sections from formalin-fixed paraffin-embedded tissue specimens were deparaffinized in xylene and rehydrated in graded alcohol with quenching of endogenous peroxidase activity by treatment with 2% H2O2 in methanol. The slides were blocked in 10% normal rabbit serum and incubated with affinity-purified anti-TERE1 (2ug/ml) for 14 hours at 4°C. After washes, the slides were incubated with biotin-conjugated rabbit IgG for 30 minutes followed by streptavidin-conjugated peroxidase and 3'3-diaminobenzidine, and counter-stained with hematoxylin. The representative anti-TERE1 staining levels were sorted into the four groups (absent, low, medium and high) based on the based on the assigned labeling index and are displayed in Fig 2. The labeling index for each core was derived by multiplying the percentage of positive cells by an intensity score (that ranged from 0 to 3+). The average value obtained from three cores was assigned as the score for that particular case.

### Expression vectors

The mammalian CMV expression plasmid pM12-NFLAG-TERE1, the pAd/CMV/V5DEST-TERE1 adenovirus, and micro RNA expression plasmid for NM_013319.1_TERE1 were previously described [25,26]. Infectious adenovirus was produced and amplified in HEK-293A cells following guidelines from Invitrogen and tittered via an anti-hexon staining procedure from Clontech to >4x10^8^ IU/ml. Infections were in the presence of 6 μg/ml polybrene and monitored via Ad*GFP* expression. All plasmids were sequenced to verify coding sequences, point and deletion mutations, reading frames, and were amplified and purified by standard techniques. Plasmids were quantified by UV and Picogreen assay (Invitrogen) and evaluated on ethidium bromide stained agarose gels.

### Transient transfection/expression analysis

Cell lines were transfected with the Nucleofector II system according to the manufacturer's protocol (Amaxa/Lonza Cologne, Germany). Expression plasmids were controlled with parallel transfections of CMV empty vector. Cells were grown in 10cm dishes and harvested after 1-3 days by washing in ice cold PBS, and scraping in the presence of protease and phosphatase inhibitors to freeze cell pellets. Preparation of whole cell extracts, BCA protein assay, SDS-PAGE and immunoblotting procedures have been described previously [32, 33].

### Cholesterol Assay

The cholesterol content of cell lysates harvested after 72 hours of transduction with Ad-*LACZ*, Ad-TERE1, or treatment with vitamin K-2 (30μM), was detected using an Amplex Red Cholesterol Assay kit relative to a dilution series of cholesterol standards as specified (Invitrogen). Lysates were prepared as previously described [32, 33].

### Cholesterol synthesis analysis by LC-MS

LnCaP cells were plated at 3e6 / 10 cm dish and when 70-80% confluent (2-3 days), serum was removed, plates washed, and cells incubated in 5% lipoprotein depleted serum with 10 mM Na ^13^C2-acetate for 72h. Cells were washed 2xPBS cold, harvested by scraping in TBS with protease inhibitors and pellets were frozen. For analysis, 300 μL of H2O with 0.1% Formic acid was added to the frozen pellets and sonicated on ice for 8' at 50% cycle time. Samples received 450 μL of methanol, were vortexed, and shaken for 30 min after adding 1.5 ml of methyl tertiary-butyl ether. The top organic layer was collected and added to injection vials. Two μLs were injected into an Agilent 1200 liquid chromatographer and analyzed by atmospheric pressure chemical ionization (APCI) with an Agilent 6410BA triple quadropole mass spectrometer. Chromatography conditions were as follows: flow rate = 0.2 ml/min, mobile phase was 80% methanol with 0.4% formic acid (FA) for 1 minute, switched to 99% methanol with 0.4% FA for 9 minutes, and re-equilibrated in 80% methanol with 0.4% FA for an additional 3 minutes. The stationary phase was a Zorbax Eclipse Plus C18, 2.1 × 100 mm, 1.8 μm particle size column. MS conditions were as follows: gas temperature = 350C, vaporizer temperature = 450C, gas flow = 8 l/min, nebulizer pressure = 60 psi, capillary voltage = 4500V, positive corona current = 4 μA, fragmenter voltage = 126V. Ions were analyzed by an MS2 scan from 368 to 400 m/z. The cholesterol peak was identified by using an internal deuterated cholesterol standard (D6-cholesterol) on an aliquot of the sample extract and performing parallel tandem MS/MS identification of the m/z transitions of 369.4 to 147.2, and 369.4 to 161.2 for unlabelled cholesterol and 375.4 to 153.2 and 375.4 to 167.2 for D6-cholesterol. Each molecule of 13C-acetate incorporated during cholesterol synthesis increases the m/z ratio of the cholesterol molecule by one, creating a series of isotopomers containing up to a maximum of 12 carbons derived from acetate C1 and 15 carbons from C2 [89]. The number of labeled carbons incorporated from ^13^C-acetate in cholesterol is proportional to the rate of cholesterol synthesis.

### RNA isolation, reverse transcription and Fluidigm RT-PCR Taqman expression assays

Cells were grown to 80% confluency, transduced with Ad-LACZ, or Ad-TERE1 and ~ 5 × 10e6 cells were lysed in 2 ml Trizol after 72 hours. Total RNA was isolated from TRIzol cell lysates (Invitrogen, Carlsbad, CA), using the Ambion Pure-Link RNA Mini kit according to procedures specified by the manufacturer (Catalog 12183-081A). RNA quantity was determined using a Nanodrop ND-1000 spectrophotometer (Thermo Scientific, Waltham, MA) and quality was assessed using an Agilent 2100 Bioanalyzer (Agilent Technologies, Santa Clara, CA). The cDNA synthesis, specific target pre-amplification, and Fluidigm RTPCR Taqman expression assays were performed using procedures recommended by ABI Biosciences and Fluidigm (40) and were performed at the UPENN Molecular Profiling Facility as previously described (24). Data was analyzed using the Fluidigm BioMark Gene Expression Data Analysis software to obtain ΔΔCt values and expressed as a ratio to the Ad-vector control to determine the fold change in gene expression.

### Taqman probes

All Taqman gene expression assays for RT-PCR were as 5´ FAM™reporter dye/3´ MGB/nonfluorescent quencher (NFQ) from Applied Biosystems/ Invitrogen. Probes were inventoried assays selected to span an exon junction and are listed: human *BACTIN* 4333762F, *GUSB* 4333767F, human *PPIA* 4333763F, *GAS6* ID: Hs00181323_m1, *AXL* ID: Hs00242357_m1, *HMGCS* ID: Hs00266810_m1, CD36, *SCARB3* ID: Hs00169627_m1, *CYP7A1* ID: Hs00167982_m1, *APOE* ID: Hs00171168_m1, *CPT1A* ID: Hs00912671_m1, *FBXW7* Hs00217794_m1, *INSIG1* ID: Hs04186616_m1, *CYP11A1* ID: Hs00167984_m1, *STAR* ID: Hs00264912_m1, *TSPO* ID: Hs00559362_m1, *SREBF1* ID: Hs01088691_m1, *SREBF2* ID: Hs01081784_m1, *FASN* ID: Hs01005622_m1, *SCD2* ID: Hs00227692_m1, SCD1 ID: Hs01682761_m1, *ST2A1* Hs00234219_m1, *FDPS* ID: Hs00266635_m1, *SXR* ID: Hs00243666_m1, *TBL2* ID: Hs00202878_m1, *UBIAD1* ID: Hs00203343_m1, *ABCG1* ID: Hs00245154_m1, *ABCA1* ID: Hs01059118_m1, *ABCB1* ID: Hs01067802_m1, *CYP3A4* ID:Hs00604506_m1, *AKR1C3* ID: Hs00366267_m1, *AKR1C2* ID: Hs00912742_m1, *AKR1C1* ID: Hs00413886_m1, *SRD5A1* ID: Hs00602694_mH, HSD3B1 ID: Hs00426435_m1, *OATP1B1* ID: Hs00272374_m1, *HMGR* ID: Hs00168352_m1, *CYP27A1* ID: Hs01026016_m1, *CYP24A1* ID: Hs00167999_m1, *CYP17A1* ID: Hs01124136_m1, *MRP2* ID: Hs01091188_m1, *UGT2B15* ID Hs03008769, *UGT2B17* ID: Hs00854486_sH.

## References

[1] Shen MM, Abate-Shen C (2010). Molecular genetics of prostate cancer: new prospects for old challenges. Genes Dev.

[2] Pelton K, Freeman MR, Solomon KR (2012). Cholesterol and prostate cancer. Curr Opin Pharmacol.

[3] Parnes HL, House MG, Tangrea JA (2013). Prostate cancer prevention: strategies for agent development. Curr Opin Oncol.

[4] Azzouni F, Mohler J (2012). Biology of castration-recurrent prostate cancer. Urol Clin North Am.

[5] Locke JA, Wasan KM, Nelson CC, Guns ES, Leon CG (2008). Androgen-mediated cholesterol metabolism in LNCaP and PC-3 cell lines is regulated through two different isoforms of acyl-coenzyme A:Cholesterol Acyltransferase (ACAT). Prostate.

[6] Leon CG, Locke JA, Adomat HH, Etinger SL, Twiddy AL, Neumann RD, Nelson CC, Guns ES, Wasan KM (2010). Alterations in cholesterol regulation contribute to the production of intratumoral androgens during progression to castration-resistant prostate cancer in a mouse xenograft model. Prostate.

[7] Dillard PR, Lin MF, Khan SA (2008). Androgen-independent prostate cancer cells acquire the complete steroidogenic potential of synthesizing testosterone from cholesterol. Mol Cell Endocrinol.

[8] Krycer JR, Kristiana I, Brown AJ (2009). Cholesterol homeostasis in two commonly used human prostate cancer cell-lines, LNCaP and PC-3. PLoS One.

[9] Montgomery RB, Mostaghel EA, Vessella R, Hess DL, Kalhorn TF, Higano CS, True LD, Nelson PS (2008). Maintenance of intratumoral androgens in metastatic prostate cancer: a mechanism for castration-resistant tumor growth. Cancer Res.

[10] Mostaghel EA, Page ST, Lin DW, Fazli L, Coleman IM, True LD, Knudsen B, Hess DL, Nelson CC, Matsumoto AM, Bremner WJ, Gleave ME, Nelson PS (2007). Intraprostatic Androgens and Androgen-Regulated Gene Expression Persist after Testosterone Suppression: Therapeutic Implications for Castration-Resistant Prostate Cancer. Cancer Research.

[11] Nelson PS (2012). Molecular states underlying androgen receptor activation: a framework for therapeutics targeting androgen signaling in prostate cancer. J Clin Oncol.

[12] Freeman MR, Solomon KR (2004). Cholesterol and prostate cancer. J Cell Biochem.

[13] Zhuang L, Kim J, Adam RM, Solomon KR, Freeman MR (2005). Cholesterol targeting alters lipid raft composition and cell survival in prostate cancer cells and xenografts. J Clin Invest.

[14] Zhuang L, Lin J, Lu ML, Solomon KR, Freeman MR (2002). Cholesterol-rich lipid rafts mediate akt-regulated survival in prostate cancer cells. Cancer Res.

[15] Li YC, Park MJ, Ye SK, Kim CW, Kim YN (2006). Elevated levels of cholesterol-rich lipid rafts in cancer cells are correlated with apoptosis sensitivity induced by cholesterol-depleting agents. Am J Pathol.

[16] Martinez-Abundis E, Garcia N, Correa F, Franco M, Zazueta C (2007). Changes in specific lipids regulate BAX-induced mitochondrial permeability transition. Febs J.

[17] Oh HY, Lee EJ, Yoon S, Chung BH, Cho KS, Hong SJ (2007). Cholesterol level of lipid raft microdomains regulates apoptotic cell death in prostate cancer cells through EGFR-mediated Akt and ERK signal transduction. Prostate.

[18] Li CG, Gruidl M, Eschrich S, McCarthy S, Wang HG, Alexandrow MG, Yeatman TJ (2008). Insig2 is associated with colon tumorigenesis and inhibits Bax-mediated apoptosis. Int J Cancer.

[19] Christenson E, Merlin S, Saito M, Schlesinger P (2008). Cholesterol effects on BAX pore activation. J Mol Biol.

[20] Decker KF, Zheng D, He Y, Bowman T, Edwards JR, Jia L (2012). Persistent androgen receptor-mediated transcription in castration-resistant prostate cancer under androgen-deprived conditions. Nucleic Acids Res.

[21] Goldstein JL, DeBose-Boyd RA, Brown MS (2006). Protein sensors for membrane sterols. Cell.

[22] Makishima M (2005). Nuclear receptors as targets for drug development: regulation of cholesterol and bile acid metabolism by nuclear receptors. J Pharmacol Sci.

[23] Wang Y, Rogers PM, Su C, Varga G, Stayrook KR, Burris TP (2008). Regulation of cholesterologenesis by the oxysterol receptor, LXRalpha. J Biol Chem.

[24] Blumberg B, Sabbagh W, Juguilon H, Bolado J, van Meter CM, Ong ES, Evans RM (1998). SXR, a novel steroid and xenobiotic-sensing nuclear receptor. Genes Dev.

[25] Zhou C, King N, Chen KY, Breslow JL (2009). Activation of PXR induces hypercholesterolemia in wild-type and accelerates atherosclerosis in apoE deficient mice. J Lipid Res.

[26] Tall AR (2008). Cholesterol efflux pathways and other potential mechanisms involved in the athero-protective effect of high density lipoproteins. J Intern Med.

[27] Zhou X, Yin Z, Guo X, Hajjar DP, Han J (2010). Inhibition of ERK1/2 and activation of liver X receptor synergistically induce macrophage ABCA1 expression and cholesterol efflux. J Biol Chem.

[28] Singh AP, Bafna S, Chaudhary K, Venkatraman G, Smith L, Eudy JD, Johansson SL, Lin MF, Batra SK (2008). Genome-wide expression profiling reveals transcriptomic variation and perturbed gene networks in androgen-dependent and androgen-independent prostate cancer cells. Cancer Lett.

[29] McGarvey TW, Nguyen T, Tomaszewski JE, Monson FC, Malkowicz SB (2001). Isolation and characterization of the TERE1 gene, a gene down-regulated in transitional cell carcinoma of the bladder. Oncogene.

[30] McGarvey TW, Nguyen T, Puthiyaveettil R, Tomaszewski JE, Malkowicz SB (2003). TERE1, a novel gene affecting growth regulation in prostate carcinoma. Prostate.

[31] Nickerson ML, Kostiha BN, Brandt W, Fredericks W, Xu KP, Yu FS, Gold B, Chodosh J, Goldberg M, Lu da W, Yamada M, Tervo TM, Grutzmacher R, Croasdale C, Hoeltzenbein M, Sutphin J (2010). UBIAD1 mutation alters a mitochondrial prenyltransferase to cause Schnyder corneal dystrophy. PLoS ONE.

[32] Fredericks WJ, McGarvey T, Wang H, Lal P, Puthiyaveettil R, Tomaszewski J, Sepulveda J, Labelle E, Weiss JS, Nickerson ML, Kruth HS, Brandt W, Wessjohann LA, Malkowicz SB (2011). The bladder tumor suppressor protein TERE1 (UBIAD1) modulates cell cholesterol: implications for tumor progression. DNA Cell Biol.

[33] Fredericks WJ, McGarvey T, Wang H, Zheng Y, Fredericks NJ, Yin H, Wang LP, Hsiao W, Lee R, Weiss JS, Nickerson ML, Kruth HS, Rauscher FJ, Malkowicz SB (2013). The TERE1 (UBIAD1) bladder tumor suppressor protein interacts with mitochondrial TBL2: regulation of trans-membrane potential, oxidative stress and SXR signaling to the nucleus. J Cell Biochem.

[34] Nickerson ML, Bosley AD, Weiss JS, Kostiha BN, Hirota Y, Brandt W, Esposito D, Kinoshita S, Wessjohann L, Morham SG, Andresson T, Kruth HS, Okano T, Dean M (2013). The UBIAD1 prenyltransferase links menaquione-4 synthesis to cholesterol metabolic enzymes. Hum Mutat.

[35] Weiss JS, Kruth HS, Kuivaniemi H, Tromp G, White PS, Winters RS, Lisch W, Henn W, Denninger E, Krause M, Wasson P, Ebenezer N, Mahurkar S, Nickerson ML (2007). Mutations in the UBIAD1 gene on chromosome short arm 1, region 36, cause Schnyder crystalline corneal dystrophy. Invest Ophthalmol Vis Sci.

[36] Nakagawa K, Hirota Y, Sawada N, Yuge N, Watanabe M, Uchino Y, Okuda N, Shimomura Y, Suhara Y, Okano T (2010). Identification of UBIAD1 as a novel human menaquinone-4 biosynthetic enzyme. Nature.

[37] Lamson DW, Plaza SM (2003). The anticancer effects of vitamin K. Altern Med Rev.

[38] Nishikawa Y, Wang Z, Kerns J, Wilcox CS, Carr BI (1999). Inhibition of hepatoma cell growth in vitro by arylating and non-arylating K vitamin analogs. Significance of protein tyrosine phosphatase inhibition. J Biol Chem.

[39] Gilloteaux J, Jamison JM, Neal DR, Loukas M, Doberzstyn T, Summers JL (2010). Cell damage and death by autoschizis in human bladder (RT4) carcinoma cells resulting from treatment with ascorbate and menadione. Ultrastruct Pathol.

[40] Vos M, Esposito G, Edirisinghe JN, Vilain S, Haddad DM, Slabbaert JR, Van Meensel S, Schaap O, De Strooper B, Meganathan R, Morais VA, Verstreken P (2012). Vitamin K2 is a mitochondrial electron carrier that rescues pink1 deficiency. Science.

[41] Nowicka B, Kruk J (2010). Occurrence, biosynthesis and function of isoprenoid quinones. Biochim Biophys Acta.

[42] Tielens AG, Rotte C, van Hellemond JJ, Martin W (2002). Mitochondria as we don't know them. Trends Biochem Sci.

[43] Ihunnah CA, Jiang M, Xie W (2011). Nuclear receptor PXR, transcriptional circuits and metabolic relevance. Biochim Biophys Acta.

[44] Zhou C, Verma S, Blumberg B (2009). The steroid and xenobiotic receptor (SXR), beyond xenobiotic metabolism. Nucl Recept Signal.

[45] Tabb MM, Sun A, Zhou C, Grun F, Errandi J, Romero K, Pham H, Inoue S, Mallick S, Lin M, Forman BM, Blumberg B (2003). Vitamin K2 regulation of bone homeostasis is mediated by the steroid and xenobiotic receptor SXR. J Biol Chem.

[46] Shearer MJ, Newman P (2008). Metabolism and cell biology of vitamin K. Thromb Haemost.

[47] Zhang B, Cheng Q, Ou Z, Lee JH, Xu M, Kochhar U, Ren S, Huang M, Pflug BR, Xie W (2010). Pregnane X Receptor As a Therapeutic Target to Inhibit Androgen Activity. Endocrinology.

[48] Amundson DM, Zhou M (1999). Fluorometric method for the enzymatic determination of cholesterol. J Biochem Biophys Methods.

[49] Fredericks WJ, Yin H, Lal P, Puthiyaveettil R, Malkowicz SB, Tomaszewski J, Fredericks NJ, Rauscher FJI, Malkowicz SB (2013). Ectopic expression of the TERE1 (UBIAD1) protein inhibits growth of Renal Clear Cell Carcinoma cells: Altered metabolic phenotype associated with reactive oxygen species, nitric oxide, and SXR target genes involved in cholesterol and lipid metabolism. Int J Oncol.

[50] Wang TT (2005). Large-Scale in Silico and Microarray-Based Identification of Direct 1,25-Dihydroxyvitamin D3 Target Genes. Molecular Endocrinology.

[51] Stewart LV, Lyles B, Lin MF, Weigel NL (2005). Vitamin D receptor agonists induce prostatic acid phosphatase to reduce cell growth and HER-2 signaling in LNCaP-derived human prostate cancer cells. J Steroid Biochem Mol Biol.

[52] Krishnan AV, Shinghal R, Raghavachari N, Brooks JD, Peehl DM, Feldman D (2004). Analysis of vitamin D-regulated gene expression in LNCaP human prostate cancer cells using cDNA microarrays. The Prostate.

[53] Landes N (2003). Homologous metabolic and gene activating routes for vitamins E and K. Molecular Aspects of Medicine.

[54] Lim YP, Huang JD (2008). Interplay of pregnane X receptor with other nuclear receptors on gene regulation. Drug Metab Pharmacokinet.

[55] Brown AJ, Jessup W (2009). Oxysterols: Sources, cellular storage and metabolism, and new insights into their roles in cholesterol homeostasis. Molecular Aspects of Medicine.

[56] Sonoda J, Chong LW, Downes M, Barish GD, Coulter S, Liddle C, Lee CH, Evans RM (2005). Pregnane X receptor prevents hepatorenal toxicity from cholesterol metabolites. Proc Natl Acad Sci U S A.

[57] Wang X, Rader DJ (2007). Molecular regulation of macrophage reverse cholesterol transport. Curr Opin Cardiol.

[58] Wang X, Collins HL, Ranalletta M, Fuki IV, Billheimer JT, Rothblat GH, Tall AR, Rader DJ (2007). Macrophage ABCA1 and ABCG1, but not SR-BI, promote macrophage reverse cholesterol transport in vivo. J Clin Invest.

[59] Patra SK (2008). Dissecting lipid raft facilitated cell signaling pathways in cancer. Biochim Biophys Acta.

[60] Swinnen JV, Brusselmans K, Verhoeven G (2006). Increased lipogenesis in cancer cells: new players, novel targets. Curr Opin Clin Nutr Metab Care.

[61] Shen MM, Abate-Shen C (2007). Pten Inactivation and the Emergence of Androgen-Independent Prostate Cancer. Cancer Research.

[62] Adam RM, Mukhopadhyay NK, Kim J, Di Vizio D, Cinar B, Boucher K, Solomon KR, Freeman MR (2007). Cholesterol sensitivity of endogenous and myristoylated Akt. Cancer Res.

[63] Montero J, Morales A, Llacuna L, Lluis JM, Terrones O, Basanez G, Antonsson B, Prieto J, Garcia-Ruiz C, Colell A, Fernandez-Checa JC (2008). Mitochondrial cholesterol contributes to chemotherapy resistance in hepatocellular carcinoma. Cancer Res.

[64] Di Vizio D, Solomon KR, Freeman MR (2008). Cholesterol and cholesterol-rich membranes in prostate cancer: an update. Tumori.

[65] Twiddy AL, Leon CG, Wasan KM (2010). Cholesterol as a Potential Target for Castration-Resistant Prostate Cancer. Pharm Res.

[66] McGarvey TW, Nguyen TB, Malkowicz SB (2005). An interaction between apolipoprotein E and TERE1 with a possible association with bladder tumor formation. J Cell Biochem.

[67] Matic M, Mahns A, Tsoli M, Corradin A, Polly P, Robertson GR (2007). Pregnane X receptor: promiscuous regulator of detoxification pathways. Int J Biochem Cell Biol.

[68] Suhara Y, Watanabe M, Motoyoshi S, Nakagawa K, Wada A, Takeda K, Takahashi K, Tokiwa H, Okano T (2011). Synthesis of new vitamin K analogues as steroid and xenobiotic receptor (SXR) agonists: insights into the biological role of the side chain part of vitamin K. J Med Chem.

[69] Suhara Y, Watanabe M, Nakagawa K, Wada A, Ito Y, Takeda K, Takahashi K, Okano T (2011). Synthesis of novel vitamin K2 analogues with modification at the omega-terminal position and their biological evaluation as potent steroid and xenobiotic receptor (SXR) agonists. J Med Chem.

[70] Nimptsch K, Rohrmann S, Kaaks R, Linseisen J (2010). Dietary vitamin K intake in relation to cancer incidence and mortality: results from the Heidelberg cohort of the European Prospective Investigation into Cancer and Nutrition (EPIC-Heidelberg). Am J Clin Nutr.

[71] Lee BH, Taylor MG, Robinet P, Smith JD, Schweitzer J, Sehayek E, Falzarano SM, Magi-Galluzzi C, Klein EA, Ting AH (2013). Dysregulation of cholesterol homeostasis in human prostate cancer through loss of ABCA1. Cancer Res.

[72] Aouabdi S, Gibson G, Plant N (2006). Transcriptional regulation of the PXR gene: identification and characterization of a functional peroxisome proliferator-activated receptor alpha binding site within the proximal promoter of PXR. Drug Metab Dispos.

[73] Ichikawa T (2006). Steroid and Xenobiotic Receptor SXR Mediates Vitamin K2-activated Transcription of Extracellular Matrix-related Genes and Collagen Accumulation in Osteoblastic Cells. Journal of Biological Chemistry.

[74] Horie-Inoue K, Inoue S (2008). Steroid and xenobiotic receptor mediates a novel vitamin K2 signaling pathway in osteoblastic cells. Journal of Bone and Mineral Metabolism.

[75] Nagaoka R, Iwasaki T, Rokutanda N, Takeshita A, Koibuchi Y, Horiguchi J, Shimokawa N, Iino Y, Morishita Y, Koibuchi N (2006). Tamoxifen activates CYP3A4 and MDR1 genes through steroid and xenobiotic receptor in breast cancer cells. Endocrine.

[76] Verma S, Tabb MM, Blumberg B (2009). Activation of the steroid and xenobiotic receptor, SXR, induces apoptosis in breast cancer cells. BMC Cancer.

[77] Trasino SE, Kim YS, Wang TT (2009). Ligand, receptor, and cell type-dependent regulation of ABCA1 and ABCG1 mRNA in prostate cancer epithelial cells. Mol Cancer Ther.

[78] Chuu CP, Hiipakka RA, Kokontis JM, Fukuchi J, Chen RY, Liao S (2006). Inhibition of tumor growth and progression of LNCaP prostate cancer cells in athymic mice by androgen and liver X receptor agonist. Cancer Res.

[79] Skowronski RJ, Peehl DM, Feldman D (1993). Vitamin D and prostate cancer: 1,25 dihydroxyvitamin D3 receptors and actions in human prostate cancer cell lines. Endocrinology.

[80] Gupta AK, Sexton RC, Rudney H (1989). Effect of vitamin D3 derivatives on cholesterol synthesis and HMG-CoA reductase activity in cultured cells. J Lipid Res.

[81] Swami S, Krishnan AV, Feldman D (2011). Vitamin D metabolism and action in the prostate: implications for health and disease. Mol Cell Endocrinol.

[82] Echchgadda I, Song CS, Roy AK, Chatterjee B (2004). Dehydroepiandrosterone sulfotransferase is a target for transcriptional induction by the vitamin D receptor. Mol Pharmacol.

[83] Gimenez-Bonafe P, Fedoruk MN, Whitmore TG, Akbari M, Ralph JL, Ettinger S, Gleave ME, Nelson CC (2004). YB-1 is upregulated during prostate cancer tumor progression and increases P-glycoprotein activity. Prostate.

[84] Penning TM, Byrns MC (2009). Steroid Hormone Transforming Aldo-Keto Reductases and Cancer. Annals of the New York Academy of Sciences.

[85] Byrns MC, Penning TM (2009). Type 5 17beta-hydroxysteroid dehydrogenase/prostaglandin F synthase (AKR1C3): role in breast cancer and inhibition by non-steroidal anti-inflammatory drug analogs. Chem Biol Interact.

[86] Wang J-H, Tuohimaa P (2007). Regulation of 17β-hydroxysteroid dehydrogenase type 2, type 4 and type 5 by calcitriol, LXR agonist and 5α-dihydrotestosterone in human prostate cancer cells. The Journal of Steroid Biochemistry and Molecular Biology.

[87] Barbier O, Belanger A (2008). Inactivation of androgens by UDP-glucuronosyltransferases in the human prostate. Best Pract Res Clin Endocrinol Metab.

[88] Kumar S, Jaiswal B, Negi S, Tyagi RK (2010). Cross-talk between androgen receptor and pregnane and xenobiotic receptor reveals existence of a novel modulatory action of anti-androgenic drugs. Biochem Pharmacol.

[89] Lindenthal B, Aldaghlas TA, Holleran AL, Sudhop T, Berthold HK, Von Bergmann K, Kelleher JK (2002). Isotopomer spectral analysis of intermediates of cholesterol synthesis in human subjects and hepatic cells. Am J Physiol Endocrinol Metab.

